# Supplementary material for: Roles and regulation of the Kunitz domain protein MLT-11 during C. elegans cuticle synthesis and molting
Source: Genetics. 2025 Dec 10;232(2):iyaf265. doi: 10.1093/genetics/iyaf265 (PMC13181417; doi:10.1093/genetics/iyaf265)
Supplement: iyaf265_Supplementary_Data [file iyaf265_supplementary_data.zip › Supplemental_Figure_Legends_GENETICS-2025-308777.pdf]

Supplemental Figure Legends

Supplementary Figure S1. Cuticle collagen and alae structure are disrupted in *mlt-11* promoter element deletion strains. a) ROL-6::mNG::3xFLAG localization in wild-type (control) and promoter deletion strains. b) BLI-1::mNG::3xFLAG localization in wild-type (control) and promoter deletion strains. c) DIC images of alae from wild-type (control), promoter deletion (peaks 3+4Δ) and knockdown (*mlt-11(RNAi))* L4+1 day worms. Images represent a minimum of 30 worms over multiple observations. Scale bars: 10 µm in (a-b), 5 µm in (c).

Figure S2. MLT-11 Kunitz domain alignment. Alignment of the ten MLT-11 Kunitz domains to Bovine Pancreatic Trypsin Inhibitor (BPTI). Positions of the six cysteine residues critical for the structure of each Kunitz domain are indicated above. Blue shading indicates conserved sequences and the histogram at the bottom depicts the degree of conservation with a consensus sequence listed below.

Figure S3. Alignment of MLT-11 homologs. MLT-11 homologs from the indicated nematode species were aligned using Clustal Omega. The length in amino acids of each homolog follows the species and homolog name. To the left and right of the alignment are amino acid positions of the end residues for each protein. Blue shading indicates conserved sequences and the histogram at the bottom depicts the degree of conservation with a consensus sequence listed below. The positions of the *C. elegans* MLT-11 signal sequence, potential furin cleavage site, thyroglobulin domain, three lustrin, and ten Kunitz domains are indicated. We truncated the extended *P. pacificus* C-terminus (amino acids 2626-3742) since no sequence aligned to it as all proteins terminated at the *C. elegans* MLT-11 stop codon. No predicted motifs are found in the *P. pacificus* C-terminus.

Figure S4. Embryonic inviability in *mlt-11* abrogated worms. The percentage of unhatched embryos from worms with either a *mlt-11* whole gene deletion or deletion of specific domains. Δ/+ worms are homozygous inviable and so were balanced and maintained as heterozygotes. These heterozygotes were plated as adults and allowed to

lay eggs for 2 hours, then removed. The resulting embryos were allowed to develop for 48 hours and then scored for hatching. Worms were scored over two biological replicates.
