# Supplementary material for: Roles and regulation of the Kunitz domain protein MLT-11 during C. elegans cuticle synthesis and molting
Source: Genetics. 2025 Dec 10;232(2):iyaf265. doi: 10.1093/genetics/iyaf265 (PMC13181417; doi:10.1093/genetics/iyaf265)
Supplement: iyaf265_Supplementary_Data [file iyaf265_supplementary_data.zip › Supplemental_Table_S1_GENETICS-2025-308777.pdf]

Table S1. Names and genotypes of strains used in this study

Name	Genotype
CB6193	*bus-8B(e2885) X* (Partridge et al. 2008)
EG7968	*unc-119(ed3) III; oxTi633 [eft-3p::tdTomato::H2B::unc-54 3'UTR + Cbr-unc-119(+)] V* (Frøkjær-Jensen et al. 2014)
IG274	*frIs7 [nlp-29p::GFP + col-12p::DsRed] IV* (Pujol et al. 2008)
JDW380	*jsTi1493 {mosL lox [wrdSi72(mlt-11p (-2.8kb:: mNeonGreen (dpi)-tbb-2 3'UTR)] FRT3::mosR} IV*
JDW383	*mlt-11(wrd78[Kunitz 2-10 deletion]), oxTi633 [eft-3p::tdTomato::H2B::unc-54 3'UTR / F46B3.7(gk5359[loxP + myo-2p::GFP::unc-54 3' UTR + rps-27p::neoR::unc-54 3' UTR + loxP]) V.*
JDW385	*him-8(e1489) IV; mlt-11(wrd80[Kunitz 7-10 deletion]), oxTi633 [eft-3p::tdTomato::H2B::unc-54 3'UTR / F46B3.7(gk5359[loxP + myo-2p::GFP::unc-54 3' UTR + rps-27p::neoR::unc-54 3' UTR + loxP]) V.*
JDW387	*mlt-11(wrd82[Kunitz 3-10 deletion]) , oxTi633 [eft-3p::tdTomato::H2B::unc-54 3'UTR / F46B3.7(gk5359[loxP + myo-2p::GFP::unc-54 3' UTR + rps-27p::neoR::unc-54 3' UTR + loxP]) V.*
JDW389	*bli-1(wrd84[linker::mNeonGreen::3xFLAG::linker])* (Johnson et al., 2023)
JDW391	*mlt-11(wrd86[C-terminal mNG::3xFLAG])V*
JDW401	*mlt-11(wrd82[Kunitz 3-10 deletion]), oxTi633 [eft-3p::tdTomato::H2B::unc-54 3'UTR / F46B3.7(gk5359[loxP + myo-2p::GFP::unc-54 3' UTR + rps-*

	27p::neoR::unc-54 3' UTR + loxP]) V. dlg-1(cp301[dlg-1::mNG-C1^3xFLAG]) X.
JDW458	*rol-6(wrd117[rol-6::C-term mNeonGreen (dpi)::3xFLAG::linker])* (Johnson et al., 2023)
JDW497	*mlt-11(wrd135[mlt-11::Kunitz 3-6 deletion]), oxTi633 [eft-3p::tdTomato::H2B::unc-54 3'UTR*
JDW503	*mlt-11(wrd137[mlt-11 thyroglobulin deletion]) , oxTi633 [eft-3p::tdTomato::H2B::unc-54 3'UTR + Cbr-unc-119(+)] V*
JDW504	*mlt-11(wrd138[mlt-11 K3-5 deletion] , oxTi633 [eft-3p::tdTomato::H2B::unc-54 3'UTR + Cbr-unc-119(+)] V*
JDW513	*mlt-11(wrd127[mlt-11 Kunitz 1-2 deletion]), oxTi633 [eft-3p::tdTomato::H2B::unc-54 3'UTR V*
JDW523	*mlt-11(wrd141[mlt-11 signal sequence deletion]) , oxTi633 [eft-3p::tdTomato::H2B::unc-54 3'UTR + Cbr-unc-119(+)]/ F46B3.7(gk5359[loxP + myo-2p::GFP::unc-54 3' UTR + rps-27p::neoR::unc-54 3' UTR + loxP]) V.*
JDW535	*jsSi1579 jsSi1706 jsSi1726 wrdSi103[loxP myo-2p::NLS::mNeonGreen, rps-0p HygR, loxP mlt-11p peak 3::pes-10 minimal promoter::mNeonGreen(dpi)::3xFLAG::PEST::tbb-2 3'UTR FRT3] II*
JDW541	*mlt-11(wrd153[mNG::3xFLAG internal]) V*
JDW551	*mlt-11(wrd158[mlt-11 peak 3Δ]), oxTi633 [eft-3p::tdTomato::H2B::unc-54 3'UTR + Cbr-unc-119(+)] V*
JDW552	*mlt-11(wrd159[mlt-11 furin site mutation]) , oxTi633 [eft-3p::tdTomato::H2B::unc-54 3'UTR + Cbr-unc-119(+)] V*
JDW558	*mlt-11(wrd153[mNG::3xFLAG internal], wrd161[mlt-11 peak 2Δ]) V*
JDW559	*mlt-11(wrd153[mNG::3xFLAG internal], wrd162[mlt-11 peak 3Δ]) V*

JDW562	*mlt-11(wrd165[Kunitz 7 deletion]), oxTi633 [eft-3p::tdTomato::H2B::unc-54 3'UTR + Cbr-unc-119(+)] V*
JDW563	*mlt-11(wrd166[Kunitz 8 deletion]), oxTi633 [eft-3p::tdTomato::H2B::unc-54 3'UTR + Cbr-unc-119(+)] V*
JDW564	*mlt-11(wrd167[Kunitz 7-8 deletion]), oxTi633 [eft-3p::tdTomato::H2B::unc-54 3'UTR + Cbr-unc-119(+)] V*
JDW565	*mlt-11(wrd168[Kunitz 10 deletion]), oxTi633 [eft-3p::tdTomato::H2B::unc-54 3'UTR + Cbr-unc-119(+)] V*
JDW566	*mlt-11(wrd169[Kunitz 9-10 deletion]), oxTi633 [eft-3p::tdTomato::H2B::unc-54 3'UTR + Cbr-unc-119(+)] V*
JDW567	*mlt-11(wrd170[Lustrin 1 deletion]), oxTi633 [eft-3p::tdTomato::H2B::unc-54 3'UTR + Cbr-unc-119(+)] V*
JDW568	*mlt-11(wrd171[Lustrin 2]), oxTi633 [eft-3p::tdTomato::H2B::unc-54 3'UTR + Cbr-unc-119(+)] V*
JDW569	*mlt-11(wrd172[Lustrin 3]), oxTi633 [eft-3p::tdTomato::H2B::unc-54 3'UTR + Cbr-unc-119(+)] V*
JDW583	*mlt-11(wrd167 wrd184 [Kunitz 7-8 deletion + Kunitz 9-10 deletion], oxTi633 [eft-3p::tdTomato::H2B::unc-54 3'UTR + Cbr-unc-119(+)] V*
JDW588	*bli-1(wrd189[bli-1::internal mNeonGreen (dpi)::3xFLAG::linker]) II, mlt-11(wrd140[mlt-11 Kunitz 1-2 deletion], oxTi633 [eft-3p::tdTomato::H2B::unc-54 3'UTR + Cbr-unc-119(+)] V*
JDW600	*rol-6(wrd198[rol-6::C-term mNeonGreen (dpi)::3xFLAG::linker]); him-8(e1489) IV; mlt-11(wrd167 wrd184 [Kunitz 7-8 deletion + Kunitz 9-10 deletion], oxTi633 [eft-3p::tdTomato::H2B::unc-54 3'UTR + Cbr-unc-119(+)] V*

JDW601	*bli-1(wrd199[bli-1::internal mNeonGreen (dpi)::3xFLAG::linker]) II; him-8(e1489) IV; mlt-11(wrd167 wrd184 [Kunitz 7-8 deletion + Kunitz 9-10 deletion], oxTi633 [eft-3p::tdTomato::H2B::unc-54 3'UTR + Cbr-unc-119(+)] V*
JDW655	*cut-2(wrd233{cut-2::C-terminal modular linker::mNG::3xFLAG::linker]) V* (Ragle et al., 2025)
JDW663	*mlt-11(wrd153[mNG::3xFLAG internal], mlt-11(wrd237[peak 4Δ]) V*
JDW666	*jsSi1579 jsSi1706 jsSi1726 wrdSi108[loxP myo-2p::NLS::mNeonGreen, rps-0p HygR, loxP mlt-11p()-2.8 kb)::mNeonGreen(dpi)::tbb-2 3'UTR FRT3]*
JDW667	*jsSi1579 jsSi1706 jsSi1726 wrdSi109[loxP myo-2p::NLS::mNeonGreen, rps-0p HygR, loxP mlt-11p peak 1::pes-10 minimal promoter::mNeonGreen(dpi)::tbb-2 3'UTR FRT3] II*
JDW668	*jsSi1579 jsSi1706 jsSi1726 wrdSi110[loxP myo-2p::NLS::mNeonGreen, rps-0p HygR, loxP mlt-11p peak 2::pes-10 minimal promoter::mNeonGreen(dpi)::tbb-2 3'UTR FRT3] II*
JDW669	*jsSi1579 jsSi1706 jsSi1726 wrdSi111[loxP myo-2p::NLS::mNeonGreen, rps-0p HygR, loxP mlt-11p peak 4::pes-10 minimal promoter::mNeonGreen(dpi)::tbb-2 3'UTR FRT3] II*
JDW670	*jsSi1579 jsSi1706 jsSi1726 wrdSi112[loxP myo-2p::NLS::mNeonGreen, rps-0p HygR, loxP mlt-11p ATAC-seq 1::pes-10 minimal promoter::mNeonGreen(dpi)::tbb-2 3'UTR FRT3] II*
JDW671	*jsSi1579 jsSi1706 jsSi1726 wrdSi113[loxP myo-2p::NLS::mNeonGreen, rps-0p HygR, loxP mlt-11p ATAC-seq 2::pes-10 minimal promoter::mNeonGreen(dpi)::tbb-2 3'UTR FRT3] II*
JDW687	*jsSi1579 jsSi1706 jsSi1726 wrdSi114[loxP myo-2p::NLS::mNeonGreen, rps-0p HygR, loxP mlt-11p(-5.3 kb)::mNeonGreen(dpi)::3xFLAG::PEST::tbb-2 3'UTR FRT3] II*

JDW696	*mlt-11(wrd153 wrd268[mNG::3xFLAG internal, peak 1Δ] V*
JDW703	*mlt-11(wrd153 wrd162 wrd 24 [mNG::3xFLAG internal, peak 3Δ, peak 4Δ] V*
JDW709	*mlt-11(wrd158 wrd252[peak 3Δ, peak 4Δ], oxTi633 [eft-3p::tdTomato::H2B::unc-54 3'UTR + Cbr-unc-119(+)] V*
JDW710	*rol-6(wrd253[rol-6::C-term mNeonGreen (dpi)::3xFLAG::linker]) II; mlt-11(wrd158[peak 3Δ]), oxTi633[eft-3p::tdTomato::H2B::unc-54 3'UTR + Cbr-unc-119(+)] V*
JDW711	*jsSi1579 jsSi1706 jsSi1726 wrdSi115[loxP myo-2p::NLS::mNeonGreen, rps-0p HygR, loxP empty control::pes-10 minimal promoter::mNeonGreen(dpi)::tbb-2 3'UTR FRT3] II*
JDW713	*bli-1(wrd254[bli-1::internal mNeonGreen (dpi)::3xFLAG::linker]) II; mlt-11(wrd158[peak 3Δ]), oxTi633 [eft-3p::tdTomato::H2B::unc-54 3'UTR + Cbr-unc-119(+)] V*
JDW715	*rol-6(wrd255[rol-6::C-term mNeonGreen (dpi)::3xFLAG::linker]) II; mlt-11(wrd158 wrd 252[peak 3Δ, peak 4Δ], oxTi633 [eft-3p::tdTomato::H2B::unc-54 3'UTR + Cbr-unc-119(+)] V*
JDW719	*jsSi1579 jsSi1706 jsSi1726 wrdSi116[loxP myo-2p::NLS::mNeonGreen, rps-0p HygR, loxP mlt-11p (-5300 to -1977 bp)::mNeonGreen (dpi)::3xFLAG::PEST::tbb-2 3'UTR FRT3] II*
JDW743	*bli-1(wrd254[bli-1::internal mNeonGreen (dpi)::3xFLAG::linker]) II; mlt-11(wrd158 wrd288[peak 3Δ, peak 4Δ]), oxTi633 [eft-3p::tdTomato::H2B::unc-54 3'UTR + Cbr-unc-119(+)] V*
JDW750	*frIs7 [nlp-29p::GFP + col-12p::DsRed] IV; mlt-11(wrd294[peak 3Δ]) V*
JDW757	*frIs7 [nlp-29p::GFP + col-12p::DsRed] IV; mlt-11(wrd294 wrd295[peak 3Δ, peak 4Δ] V*
JDW796	*jsTi1493 {mosL loxP [wrdSi70(mlt-11p (-2.8 kb)::nuc-1::mCherry-tbb-2 3'UTR)] FRT3::mosR} IV ; mlt-11(wrd153[mNG::3xFLAG internal]) V*
JDW807	*mlt-11(wrd333[exon 10-11 deletion]), oxTi633 [eft-3p::tdTomato::H2B::unc-54 3'UTR + Cbr-unc-119(+)] V*

JDW913	*rol-6(wrd385[rol-6::mNG::3xFLAG]), dpy-10(syb4556 wrd383[dpy-10::mScarlet]) II*
JDW916	*unc-119(ed3) III; mlt-11(wrd388[mlt-11 deletion between Kunitz 8 and 9]), oxTi633 [eft-3p::tdTomato::H2B::unc-54 3'UTR + Cbr-unc-119(+)] V*
JDW917	*bli-1(wrd84[bli-1::internal modular linker::mNG::3xFLAG::linker]) II; mlt-11(wrd333[exon 10-11 deletion left roller]) , oxTi633 [eft-3p::tdTomato::H2B::unc-54 3'UTR + Cbr-unc-119(+)] V*
JDW929	*mlt-11(wrd397[mlt-11 whole gene deletion]), oxTi633 [eft-3p::tdTomato::H2B::unc-54 3'UTR + Cbr-unc-119(+)] / F46B3.7(gk5359[loxP + myo-2p::GFP::unc-54 3' UTR + rps-27p::neoR::unc-54 3' UTR + loxP]) V*
N2	Wild type
NM5179	*jsTi1493 [LoxP::mex-5p::FLP:SL2::mNeonGreen::rpl-28p::FRT::GFP::his-58::FRT3]* IV (Nonet 2020)
NM5548	*jsSi1579 jsSi1706 jsSi1726[loxP myo-2p FRT nlsCyOFP myo-2 3' mex-5p FLP D5 glh-2 3' FRT3] II* (Nonet 2023)
