# Supplementary figures and images for: Roles and regulation of the Kunitz domain protein MLT-11 during C. elegans cuticle synthesis and molting
Source: Genetics. 2025 Dec 10;232(2):iyaf265. doi: 10.1093/genetics/iyaf265 (PMC13181417; doi:10.1093/genetics/iyaf265)

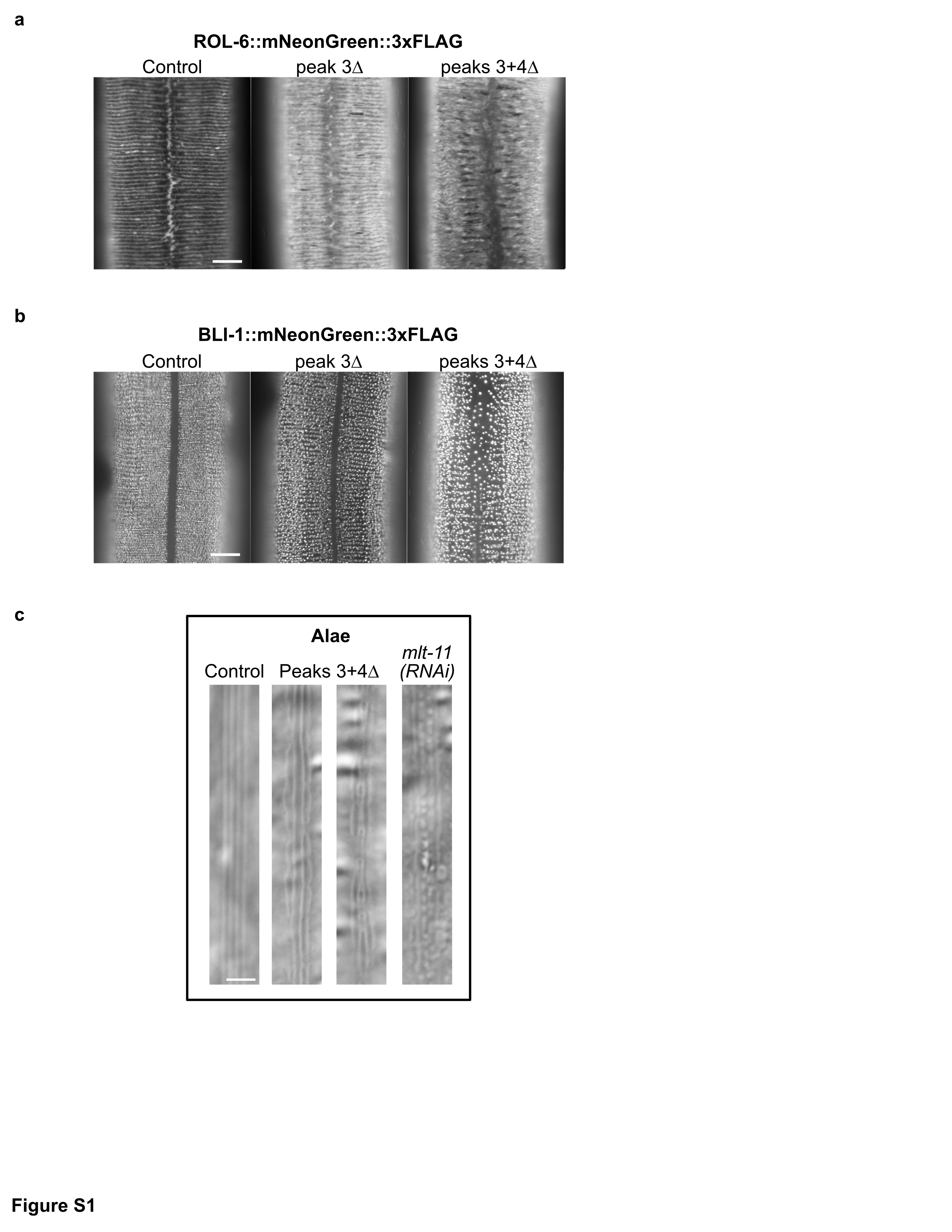

Supplement: iyaf265_Supplementary_Data [file iyaf265_supplementary_data.zip › Supplemental_Figure_S1_GENETICS-2025-308777.tif]

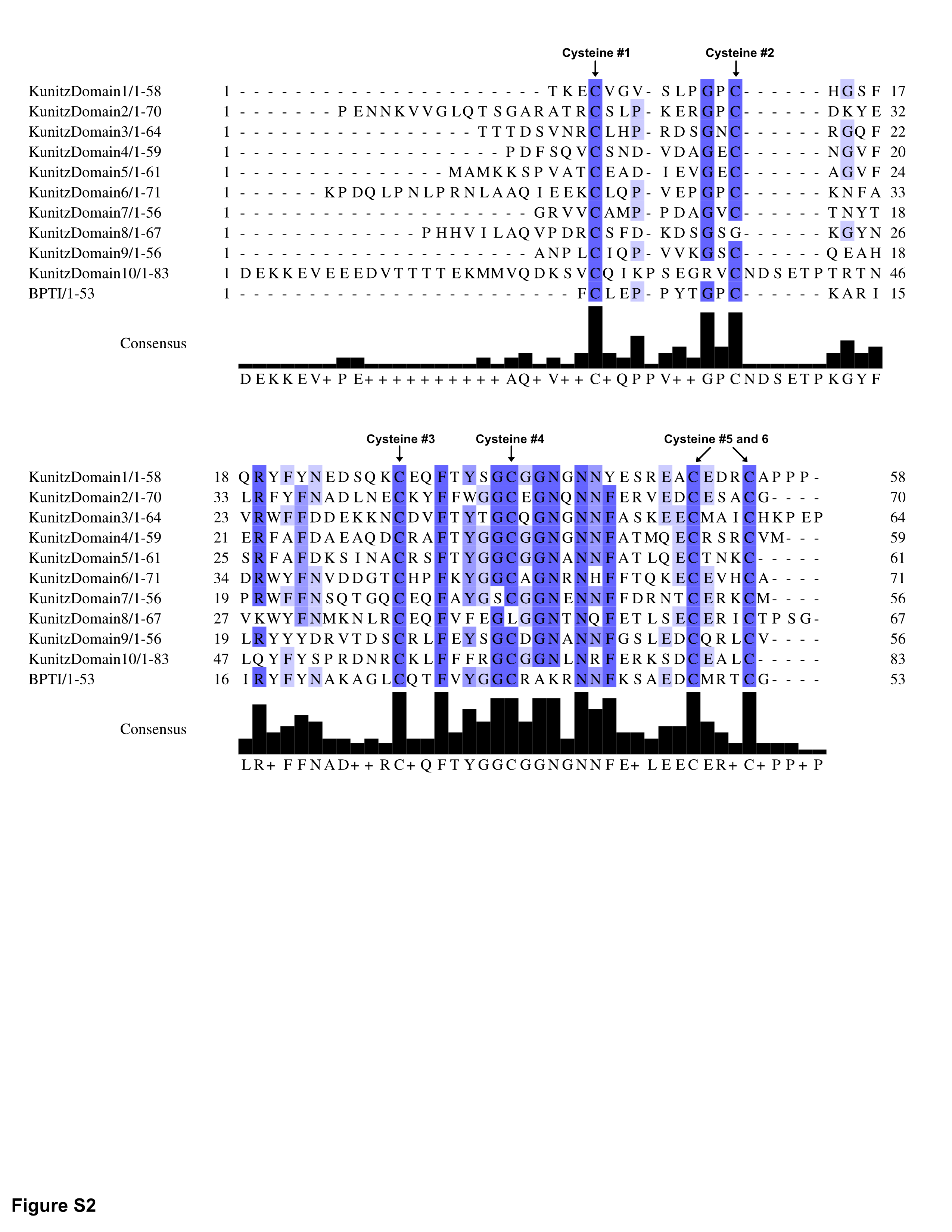

Supplement: iyaf265_Supplementary_Data [file iyaf265_supplementary_data.zip › Supplemental_Figure_S2_GENETICS-2025-308777.tif]

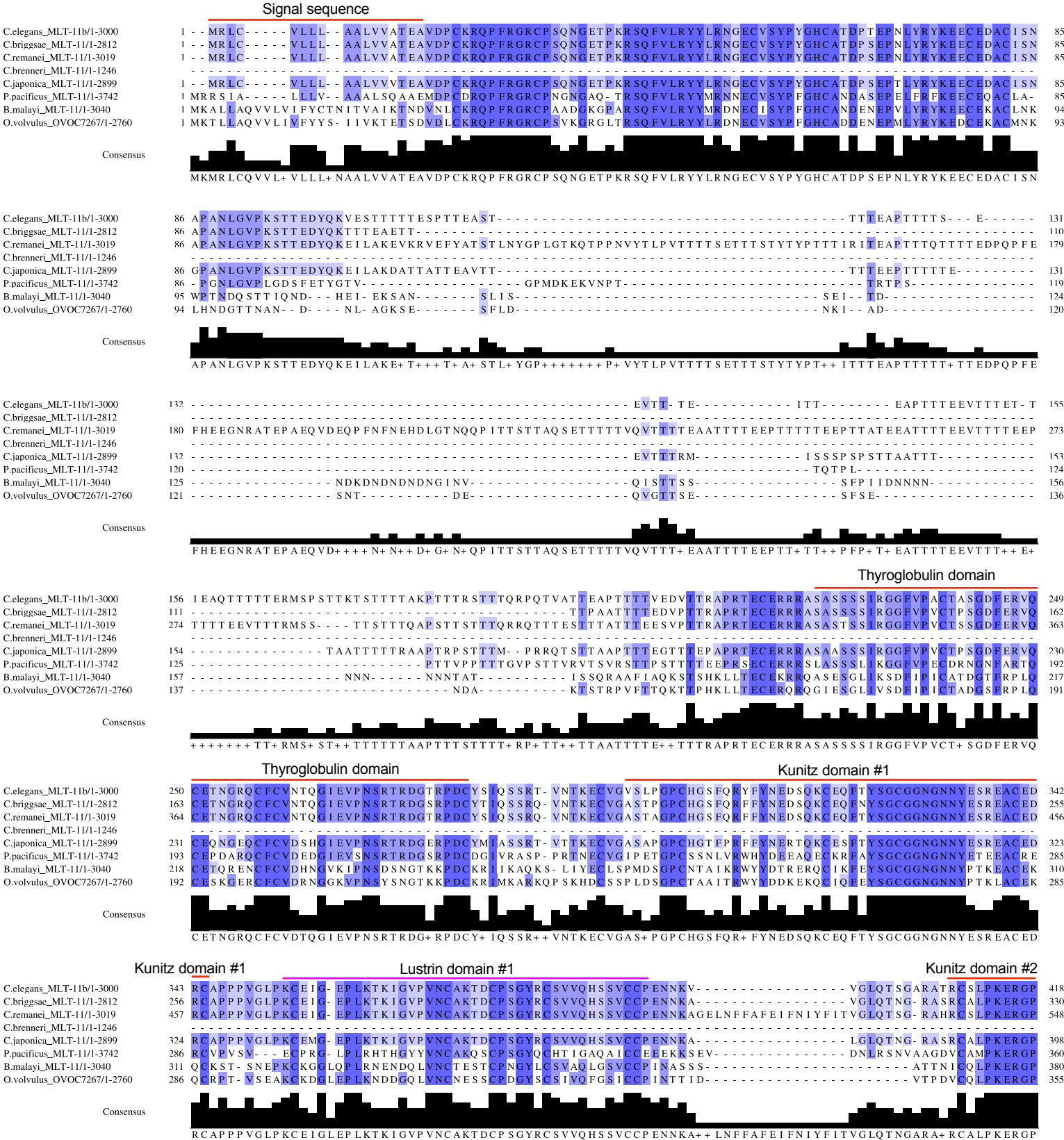

Figure S3

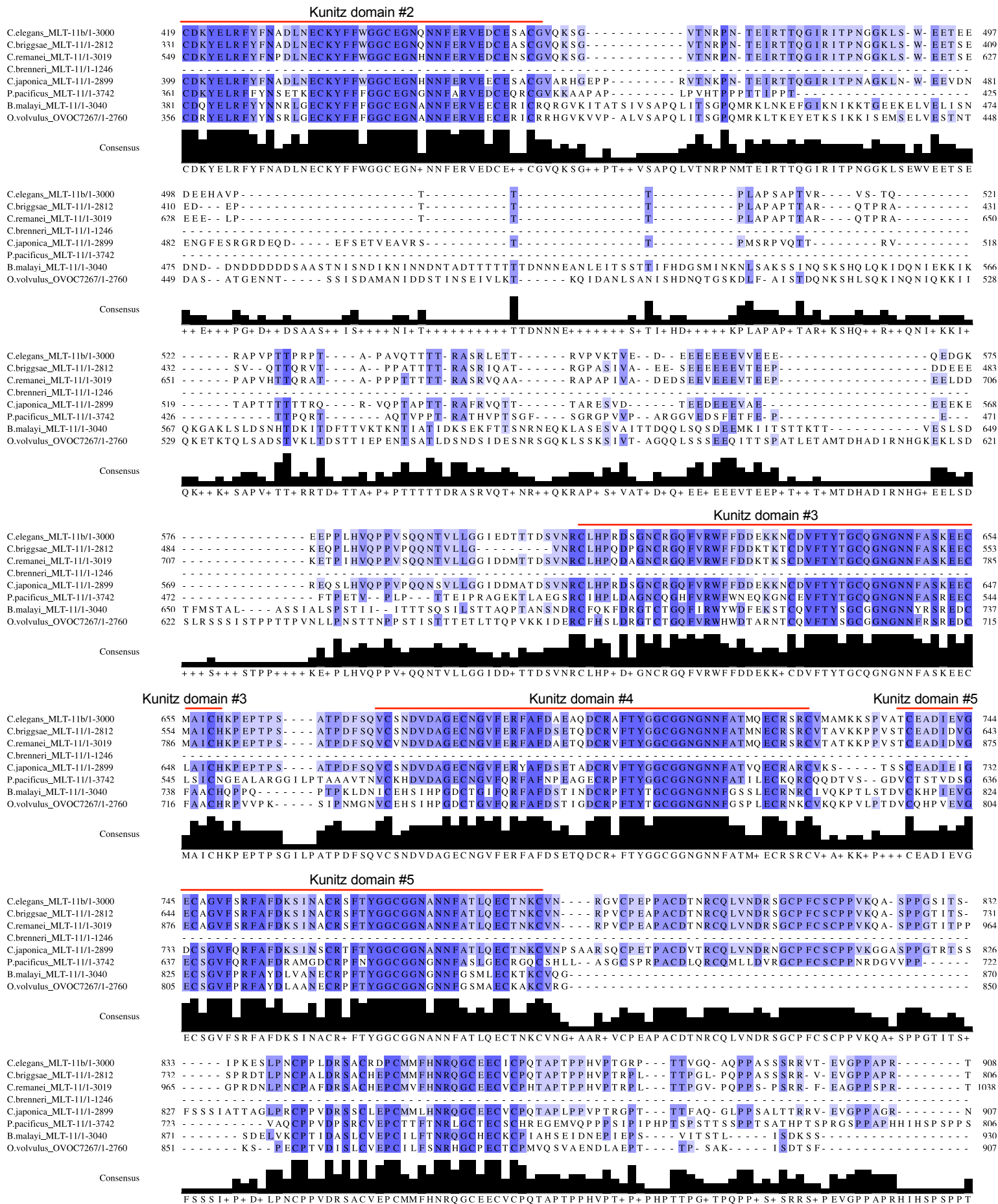

Figure S3

## Kunitz domain #6

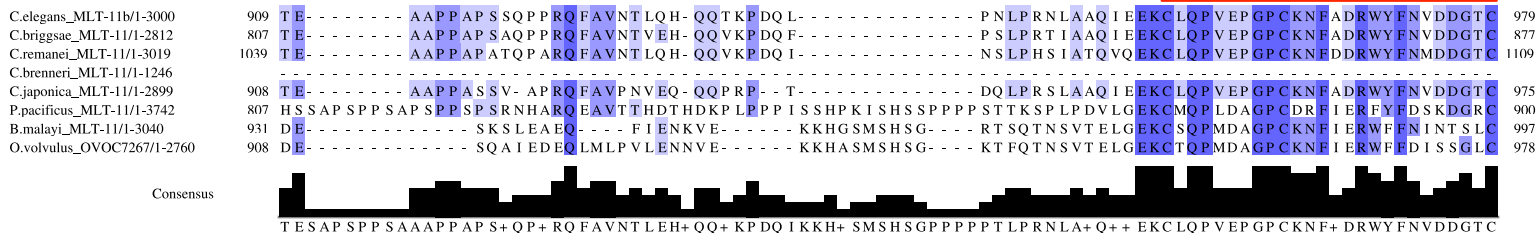

## Kunitz domain #6

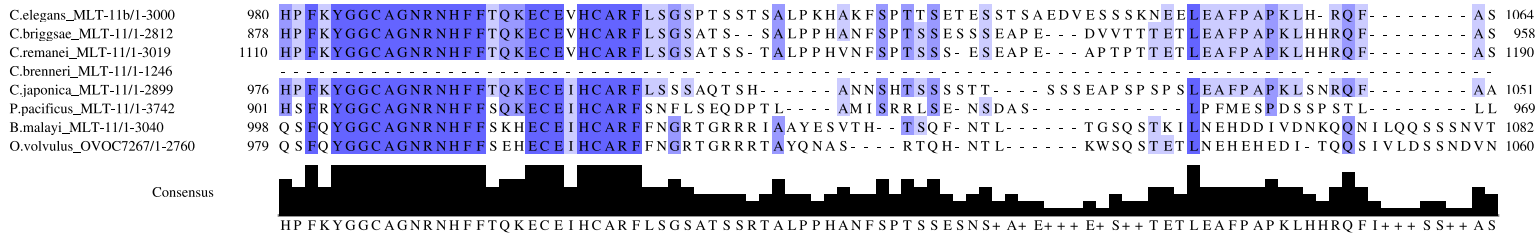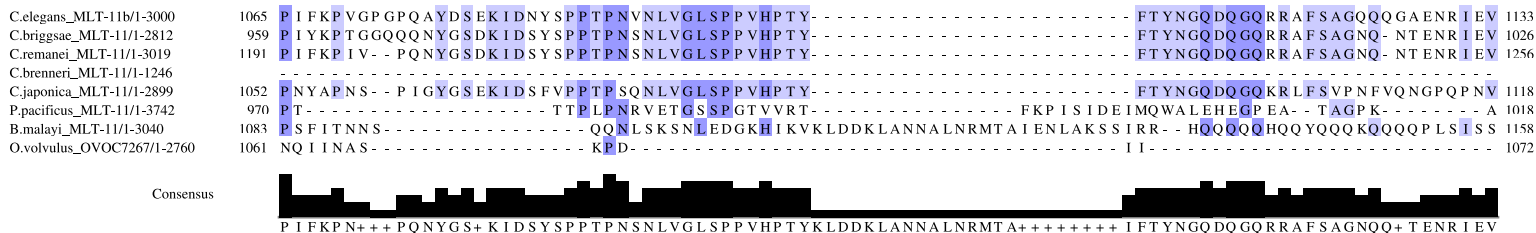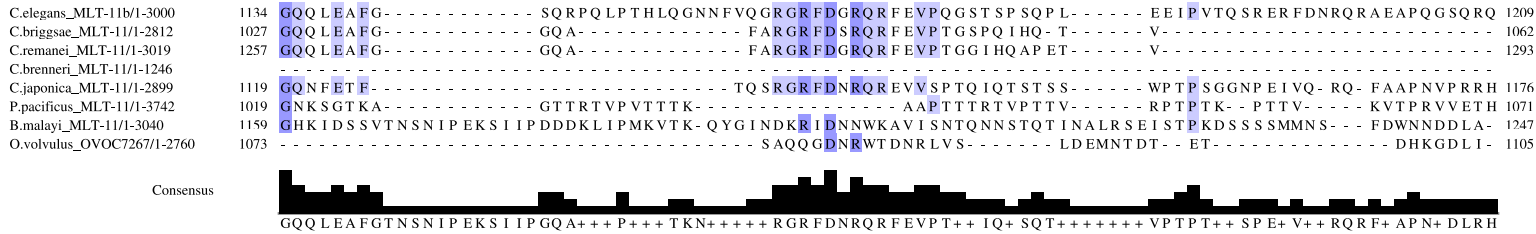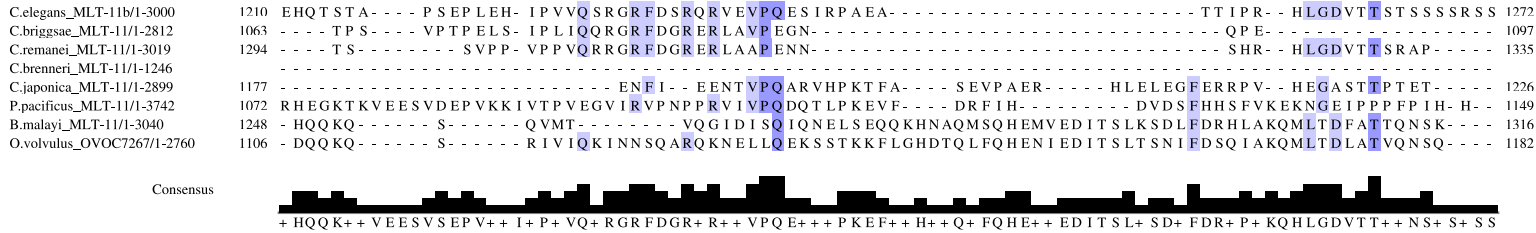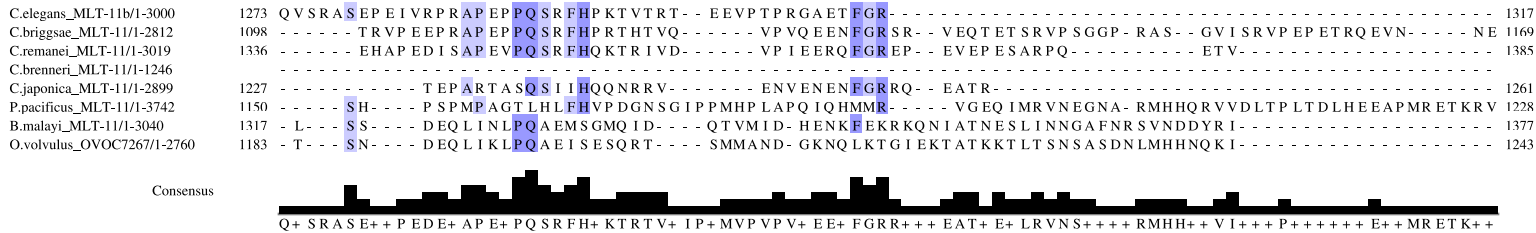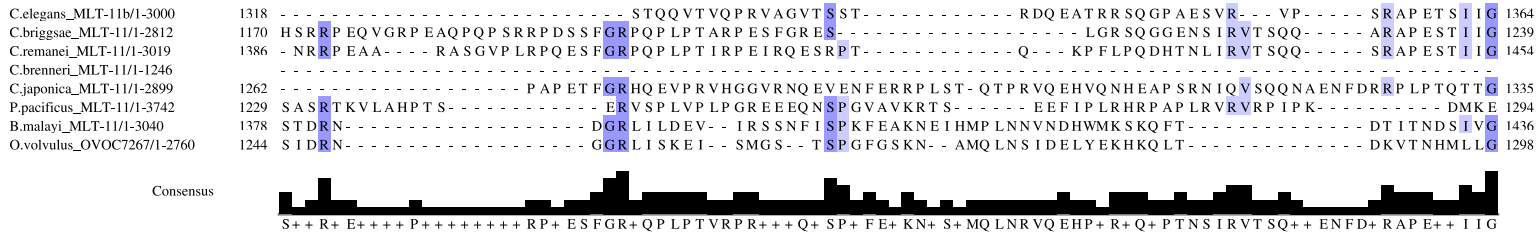

Figure S3

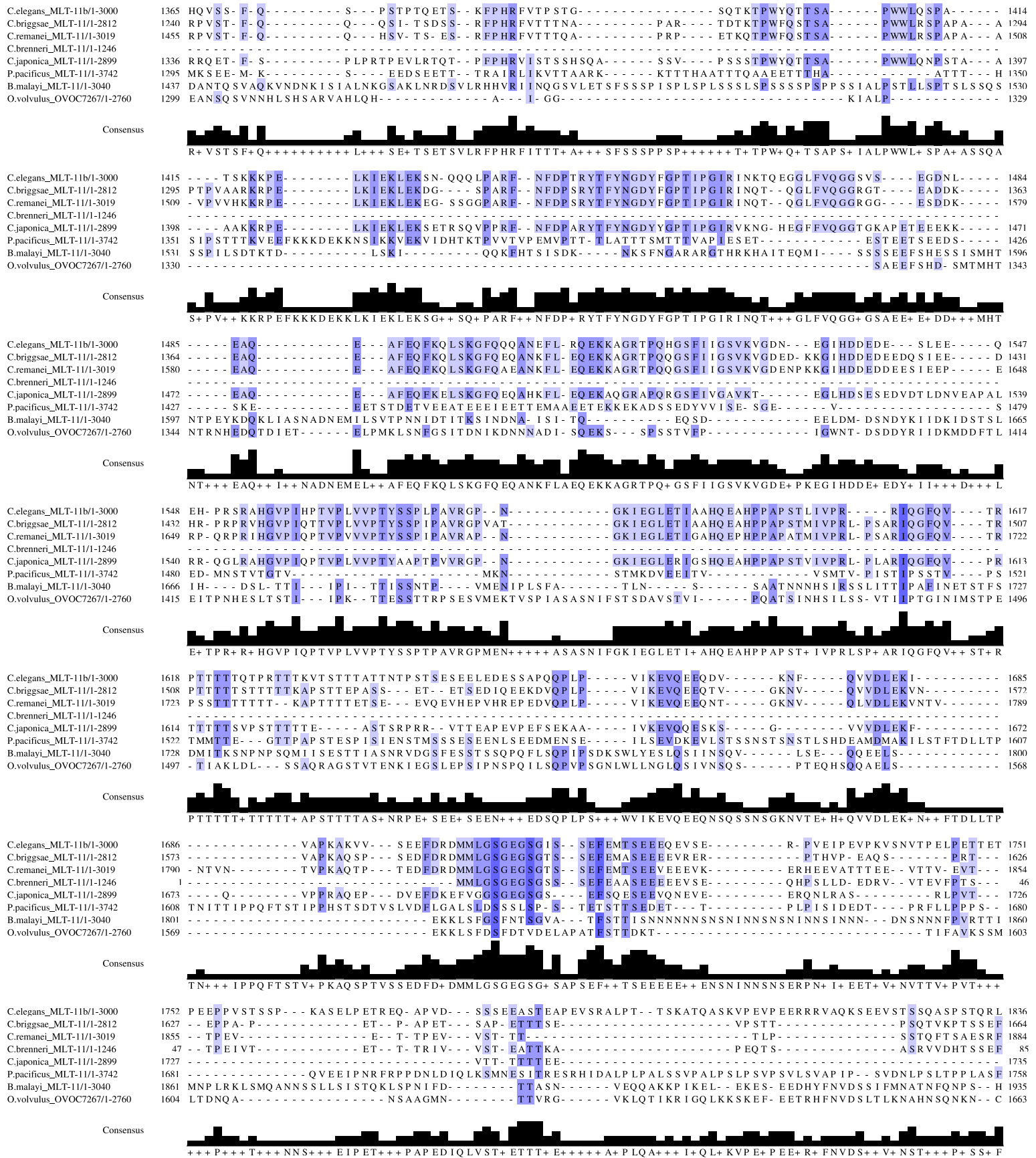

Figure S3

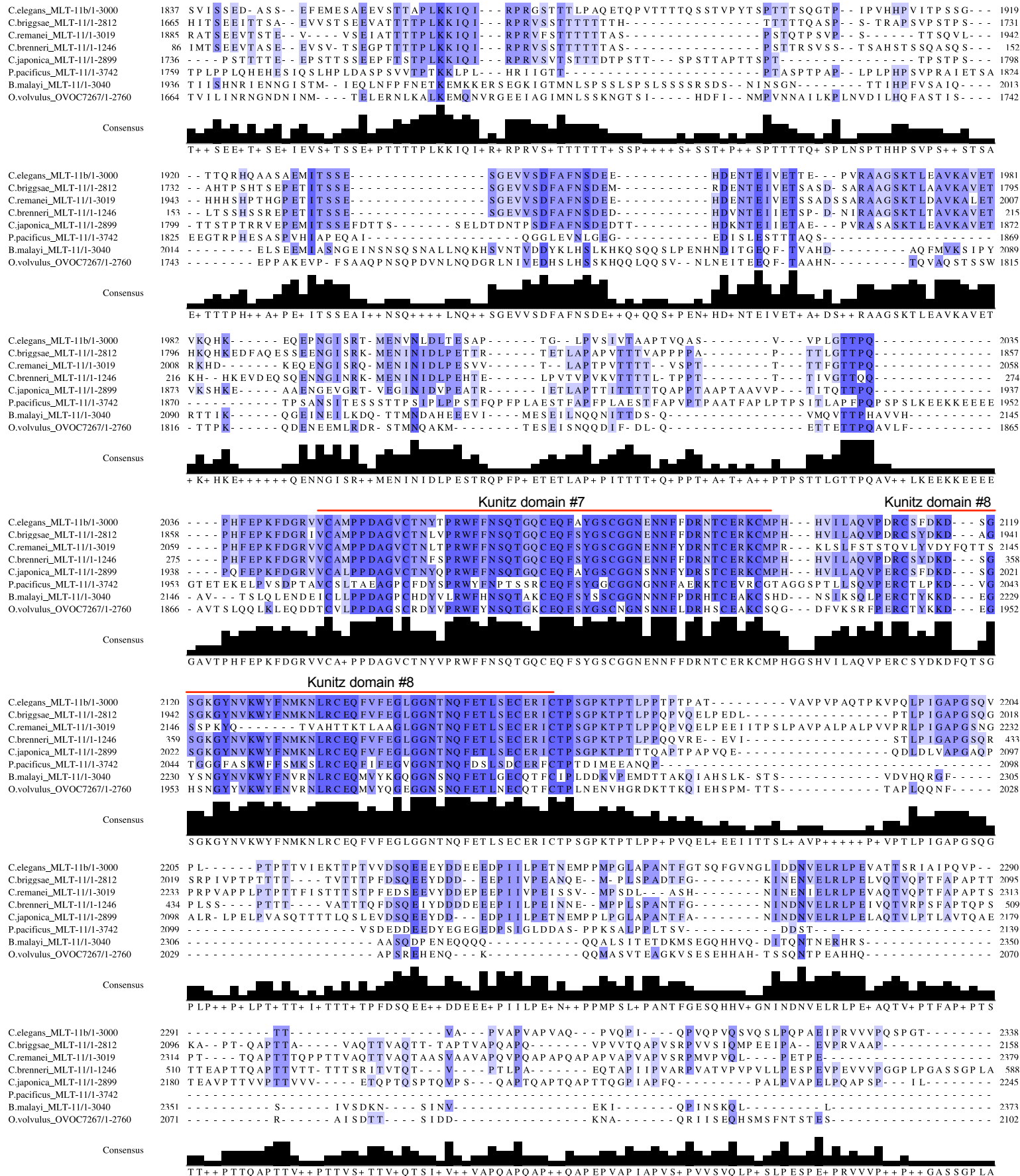

Figure S3

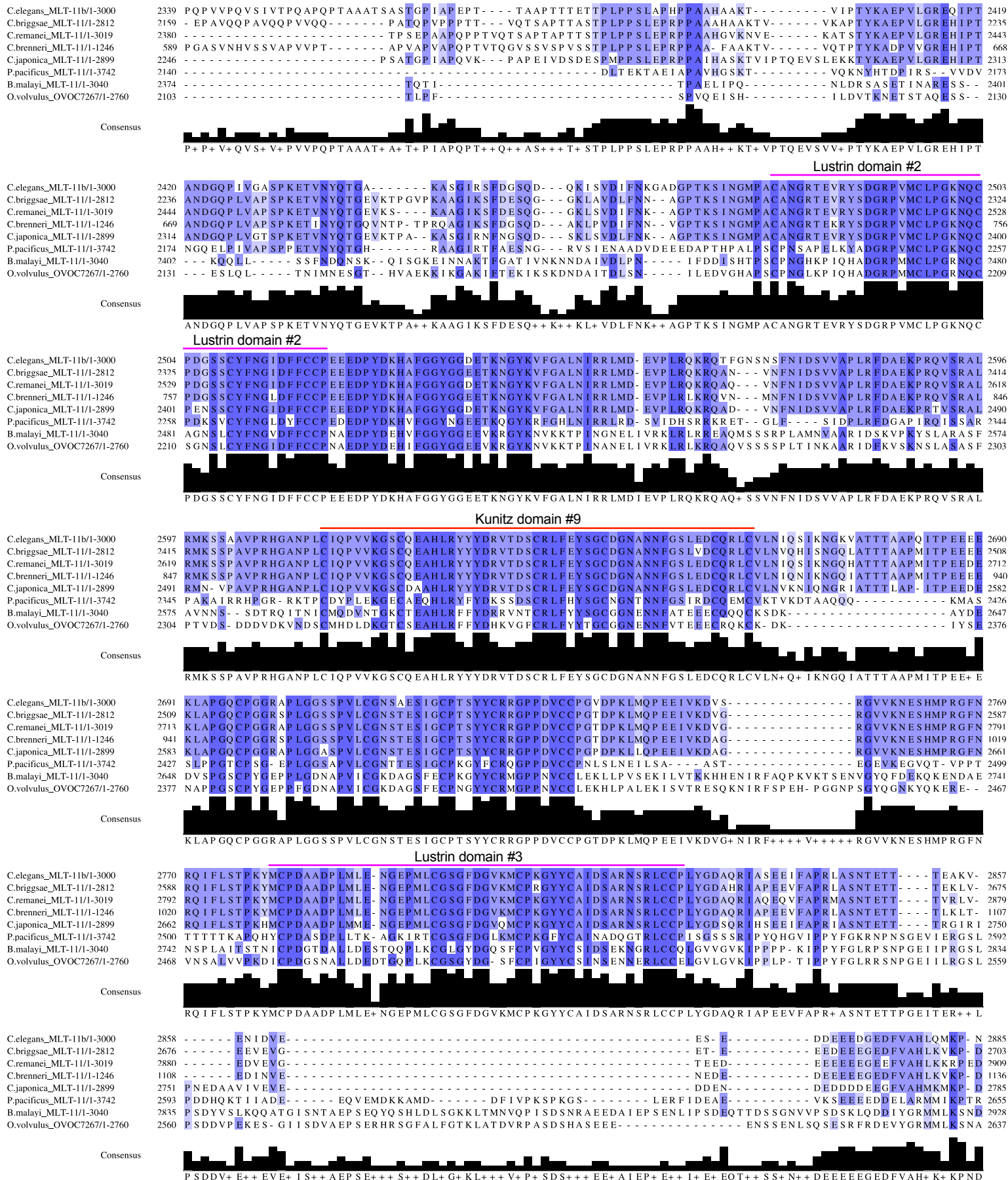

Figure S3

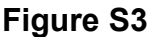

Supplement: iyaf265_Supplementary_Data [file iyaf265_supplementary_data.zip › Supplemental_Figure_S3_GENETICS-2025-308777.pdf]

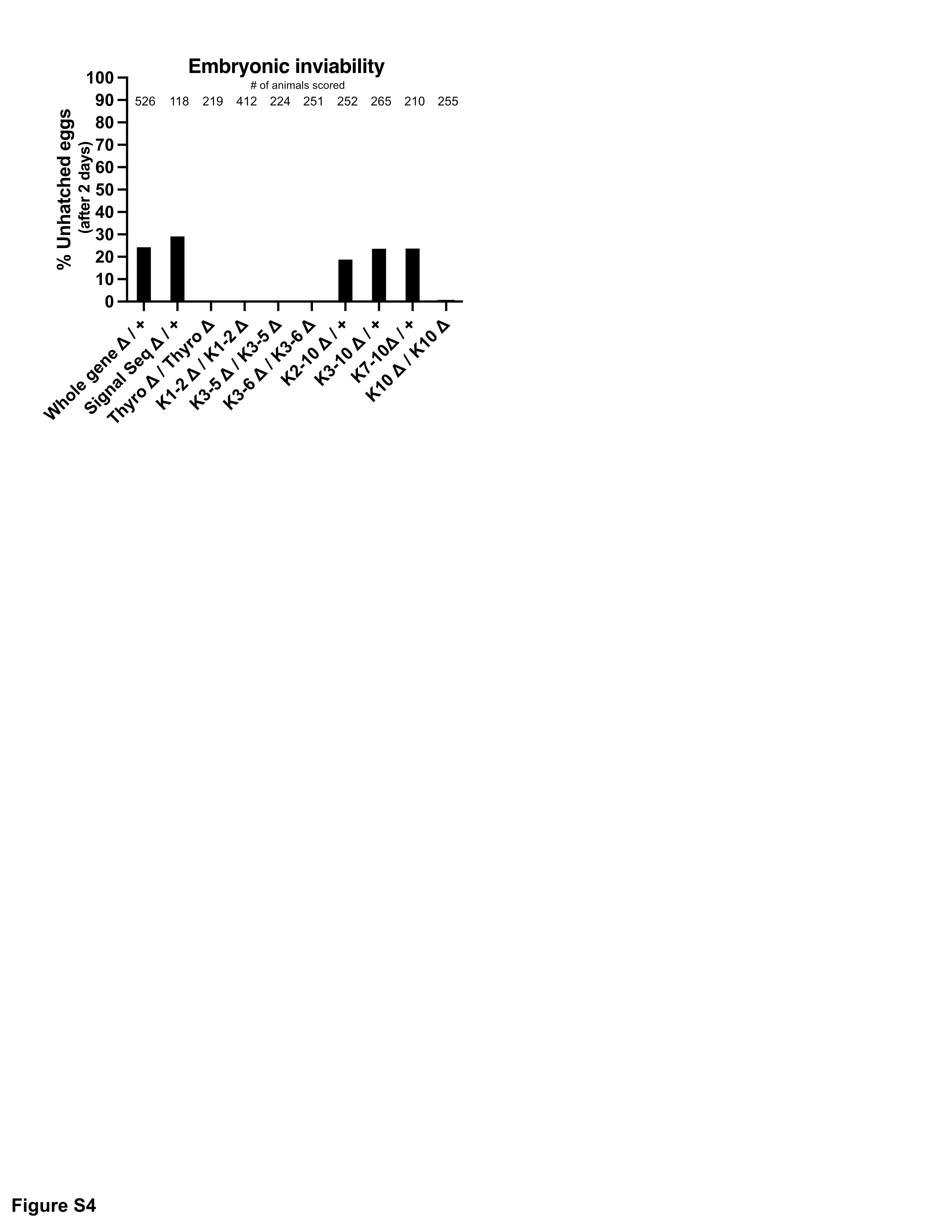

Supplement: iyaf265_Supplementary_Data [file iyaf265_supplementary_data.zip › Supplemental_Figure_S4_GENETICS-2025-308777.tif]
